# The GhWRKY70-*GhAOS1* Axis Integrates Jasmonate Pathway Signaling to Regulate Cotton Immunity Against *Verticillium dahliae*

**DOI:** 10.3390/ijms27114713

**Published:** 2026-05-23

**Authors:** Huiting Weng, Chao Zhang, Suoxian Li, Huiming Guo, Hongmei Cheng, Wenfang Guo, Xiaofeng Su

**Affiliations:** 1 State Key Laboratory of North China Crop Improvement and Regulation, College of Life Science, Hebei Agricultural University, Baoding 071001, China; wht_bio@163.com (H.W.); zhang_chao@hebau.edu.cn (C.Z.); 2 National Key Laboratory of Agricultural Microbiology, Biotechnology Research Institute, Chinese Academy of Agricultural Sciences, Beijing 100081, China; lsxdwyyx2023@163.com (S.L.); guohuiming@caas.cn (H.G.); chenghongmei@caas.cn (H.C.); 3 Academician Workstation, National Nanfan Research Institute, Chinese Academy of Agricultural Sciences, Sanya 572024, China

**Keywords:** Verticillium wilt, jasmonic acid, *GhAOS1*, GhWRKY70

## Abstract

Verticillium wilt (VW), caused by the soil-borne phytopathogen *Verticillium dahliae*, is a devastating vascular disease that severely threatens global cotton production and causes substantial economic losses. Jasmonic acid (JA) signaling plays a crucial role in plant innate immunity; however, the molecular mechanisms governing JA biosynthesis during cotton defense responses to *V. dahliae* infection remain largely elusive. In this study, we identified that *GhAOS1* (allene oxide synthase 1), a key rate-limiting enzyme-encoding gene in the JA biosynthetic pathway, was rapidly and significantly induced by *V. dahliae* infection and exclusively localized in chloroplasts. Functional analysis in *GhAOS1*-silenced cotton and overexpressing *Arabidopsis* plants demonstrated that *GhAOS1* positively regulates resistance to *V. dahliae*. Transcriptome analysis of *GhAOS1*-silenced cotton plants showed that DEGs are significantly enriched in phenylpropanoid biosynthesis, flavonoid biosynthesis, and α-linolenic acid metabolism pathways. Consistent with these findings, silencing *GhAOS1* significantly reduced endogenous JA levels and suppressed the expression of defense-related genes and JA biosynthetic genes in cotton. Furthermore, we identified that the transcription factor GhWRKY70 directly binds to the W-box cis-acting element in the *GhAOS1* promoter through Y1H, LUC, and EMSA, which activated *GhAOS1* transcription. Silencing *GhWRKY70* in cotton significantly enhanced plant susceptibility to *V. dahliae* and suppressed the expression of JA signaling pathway-related genes. Collectively, our results elucidate that *GhWRKY70* positively regulates cotton resistance to VW by activating *GhAOS1*-mediated JA biosynthesis, revealing a novel GhWRKY70-*GhAOS1* regulatory module that integrates JA signaling to coordinate cotton immune responses against *V. dahliae*. This study provides new insights into the molecular mechanism of JA-mediated defense and offers potential targets for molecular breeding of VW-resistant cotton.

## 1. Introduction

Cotton is one of the most important economic crops worldwide and occupies a strategic position in global agriculture [1]. Verticillium wilt (VW) of cotton is a highly destructive soil-borne vascular disease caused by *Verticillium dahliae* and *V. albo-atrum*. After invading through the roots, the pathogens colonize the vascular tissues, leading to systemic infection in the host plant. Typical symptoms include leaf wilting and vascular browning, and in severe cases, complete plant death [2]. The occurrence of VW poses a serious threat to the cotton industry and results in substantial economic losses. According to incomplete statistics, cotton yield can be reduced by 10–30% in regions with high disease incidence, and in severe cases, losses can exceed 80%, with complete crop failure occurring in some fields [3]. Genetic engineering through the expression of key disease resistance genes has proven to be an effective strategy for developing transgenic plants with enhanced resistance to pathogens [4,5]. Therefore, it is crucial to understand the underlying physiology and molecular mechanisms in cotton defense against *V. dahliae* infection.

Jasmonic acid (JA) and its derivatives, collectively referred to as jasmonates (JAs), function as key stress-responsive phytohormones and play essential roles in plant defense against both biotic and abiotic stresses [6]. Allene oxide synthase (AOS), also known as cytochrome P450 CYP74, is a key rate-limiting enzyme in the JA biosynthesis pathway [7]. As the enzyme catalyzing the first irreversible step in JA biosynthesis, AOS is rapidly induced upon PRR-mediated recognition of pathogen-associated molecular patterns (PAMPs) or damage-associated molecular patterns (DAMPs), leading to increased accumulation of JA and JA-Ile and activation of JA-dependent plant immune responses [8,9,10,11,12]. Consistently, transgenic potato plants lacking *St13-AOS* exhibit significantly reduced JA levels, increased susceptibility to soft rot caused by fungal pathogens [13]. In *Arabidopsis*, *AOS* overexpression delays pathogen infection and maintains plant growth under stress, accompanied by increased expression of JA- and salicylic acid-related defense genes [14]. These findings indicate that AOS plays a pivotal role in regulating JA biosynthesis and plant immunity. However, the upstream regulatory mechanisms controlling AOS expression and the downstream components mediating AOS-dependent defense responses in cotton remain largely unknown.

WRKY transcription factors are widely involved in plant growth, development, and stress adaptation [15,16]. The WRKY family of transcription factors is defined by the presence of a conserved WRKYGQK amino acid sequence and can be subdivided into three groups [17,18]. Numerous studies have demonstrated that WRKY TFs function as key regulators of JA-mediated defense signaling, particularly through their interactions with jasmonate ZIM-domain (JAZ) and VQ proteins, which enable them to fine-tune JA signaling and orchestrate plant responses to environmental stresses [19]. In *Arabidopsis*, WRKY33 and WRKY57 competitively bind to the VQ motifs of SIB1 and SIB2, thereby regulating the expression of JAZ1 and JAZ5 and fine-tuning JA signaling and resistance to the necrotrophic pathogen *Botrytis cinerea* [20]. In soybean, GmWRKY40 directly binds to and represses the promoter of *GmJAZ1*, thereby relieving the suppression of JA signaling, leading to increased endogenous JA levels and enhanced resistance to *Phytophthora sojae* [21]. SlWRKY31, along with its interaction partner SlVQ15, positively regulates disease resistance to *B. cinerea*, while SlJAZ11 disrupts the interaction and promotes SlWRKY31 transactivation activity [22]. Several WRKY TFs have been shown to positively regulate resistance to *V. dahliae* in cotton [23,24,25,26]. However, the roles and underlying mechanisms of WRKY transcription factors in regulating JA signaling during cotton resistance to VW remain poorly understood.

In our previous study, integrated transcriptomic analyses of cotton infected with *V. dahliae* identified *GhAOS1*, a key gene involved in JA biosynthesis, as a candidate regulator of VW resistance [27]. Building on this finding, the present study systematically investigated the function and regulatory mechanism of *GhAOS1* in cotton immunity. We show that *GhAOS1* localizes in chloroplasts and is transcriptionally induced upon *V. dahliae* infection. Functional analyses using VIGS in cotton and heterologous overexpression in *Arabidopsis* confirm that *GhAOS1* positively regulates resistance to *V. dahliae*. Transcriptome and qRT-PCR analyses further reveal that *GhAOS1* modulates the expression of defense-related and JA biosynthesis genes, indicating that its resistance function is mediated through the JA signaling pathway. Moreover, we demonstrate that *GhAO*S*1* expression is directly activated by the transcription factor GhWRKY70, which regulates JA signaling and enhances cotton resistance to VW. These findings uncover a GhWRKY70-*GhAOS1* regulatory module and provide new insights into the molecular mechanisms underlying JA-mediated defense and VW resistance in cotton.

## 2. Results

### 2.1. Response to V. dahliae and Identification of GhAOS1

**Figure 1:**
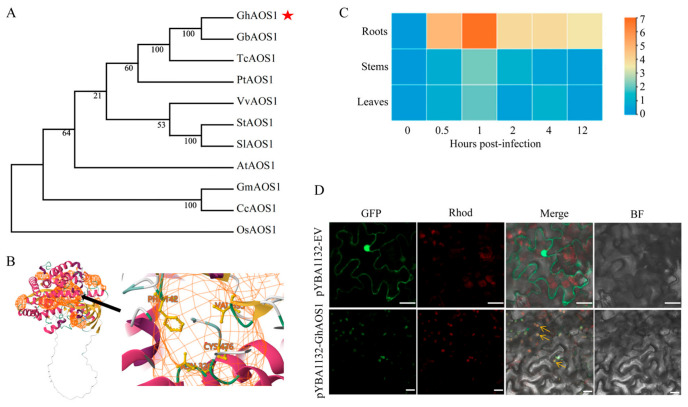
Characterization of GhAOS1 expression, protein structure, and subcellular localization. (**A**) Phylogenetic tree of AOS1 proteins from *Gossypium hirsutum* (Gh, marked with red star), *Gossypium barbadense* (Gb), *Theobroma cacao* (Tc), *Populus trichocarpa* (Pt), *Vitis vinifera* (Vv), *Solanum tuberosum* (St), *Solanum lycopersicum* (Sl), *Arabidopsis thaliana* (At), *Glycine max* (Gm), *Cajanus cajan* (Cc), and *Oryza sativa* (Os). (**B**) Prediction of the tertiary structure and active site of the GhAOS1 protein. (**C**) Temporal expression profiles of *GhAOS1* in roots, stems, and leaves of cotton following *V. dahliae* infection at 0, 0.5, 1, 2, 4, and 12 hpi. Data are shown as mean ± SE (*n* = 3). (**D**) Subcellular localization of GhAOS1. BF, the bright field; GFP, green fluorescent channel; Rhod, chloroplast autofluorescence channel. Merge, three-channel merge. Scale bars = 20 μm.

Phylogenetic analysis revealed that GhAOS1 from *G. hirsutum* was the closest ortholog of GbAOS1 from *Gossypium barbadense* (Figure 1A). Elucidating the tertiary structure and active sites of GhAOS1 protein is crucial for understanding the molecular mechanism underlying its catalysis of JA biosynthesis. Homology modeling of GhAOS1 was performed using the PlayMolecule online server, revealing that the tertiary structure is predominantly composed of α-helices (38.43%), extended strands (9.18%), and random coils (52.39%). Further analysis identified multiple potential active pockets, among which pocket 1, predicted to possess the highest activity, contains key residues on chain A: phenylalanine (F142), asparagine (N326), valine (V393), and cysteine (C476). These residues may play important roles in maintaining catalytic activity and participating in substrate binding (Figure 1B). Temporal expression profiles showed that GhAOS1 expression was slightly induced at 0.5 hpi and reached peak expression at 1 hpi in roots. (Figure 1C). Subcellular localization confirmed that GhAOS1 was localized in the Chloroplast (Figure 1D). Collectively, these findings implied that *GhAOS1* may play a critical role in cotton defense against *V. dahliae*.

### 2.2. GhAOS1 Positively Regulated Cotton Resistance to V. dahliae

**Figure 2:**
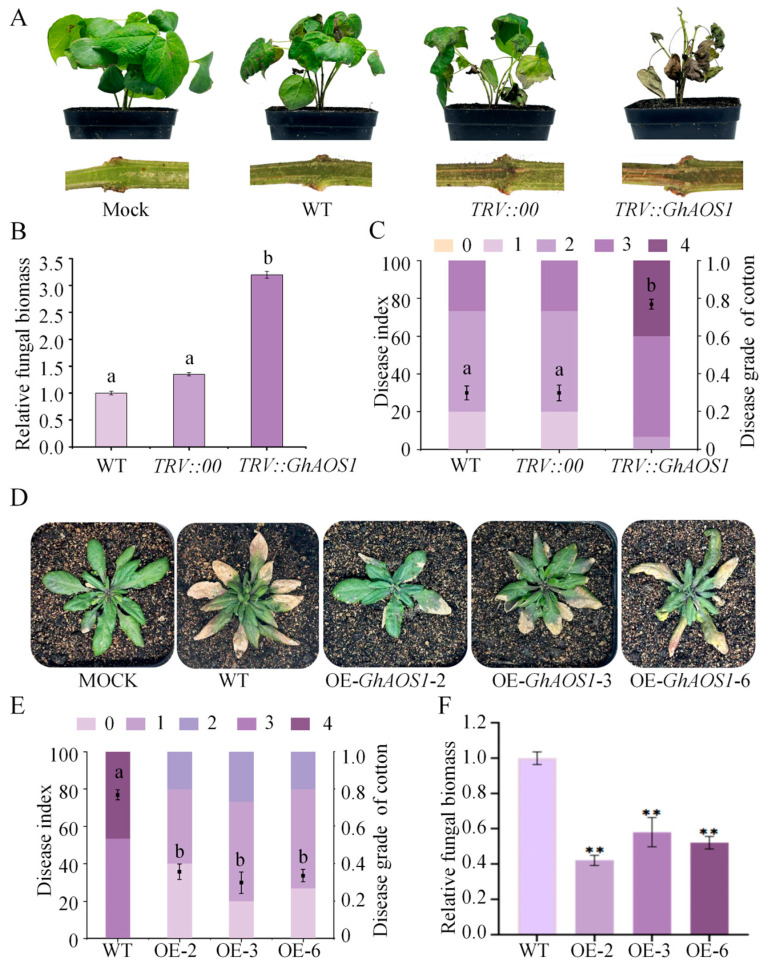
*GhAOS1* positively regulates cotton resistance to *V. dahliae*. (**A**) Disease symptoms of cotton plants injected with TRV::*00* (control) or TRV::*GhAOS1* at 15 dpi with *V. dahliae*. (**B**) Relative fungal biomass in cotton stems quantified by the ratio of *VdITS/GhUBQ7*. (**C**) DI and disease grade distribution of cotton plants at 15 dpi. Data represent means ± SE (*n* > 25). (**D**) Disease symptoms of *Arabidopsis* plants overexpression (OE-*GhAOS1*-2/3/6) at 14 dpi with *V. dahliae*. (**E**) DI and disease grade distribution of *Arabidopsis* plants at 15 dpi with *V. dahliae*. Data represent means ± SE (*n* > 25). (**F**) Fungal biomass analysis of WT and transgenic *Arabidopsis* lines (OE-*GhAOS1*-2/3/6) at 14 dpi. Different letters indicate significant differences at *p* < 0.05 based on one-way ANOVA followed by Tukey’s test. Asterisks indicate significant differences based on Student’s *t*-test (**, *p* < 0.01).

To investigate the function of *GhAOS1* in cotton defense against *V. dahliae*, cotton seedlings at the three-cotyledon stage were infiltrated with *Agrobacterium* suspensions carrying TRV::*00* (negative control), TRV::*GhAOS1*, or TRV::*CLA1* (positive control). When obvious albino phenotypes appeared on the true leaves of plants treated with TRV::*CLA1*, total RNA was extracted from the leaves for qRT-PCR analysis. The results showed that the expression level of *GhAOS1* in the TRV::*GhAOS1*-treated group was significantly reduced by approximately 85% compared with the empty vector (TRV::*00*) control, indicating that the gene silencing efficiency met the requirements for subsequent experiments (Figure S1A,B). Subsequently, the silenced plants and control plants were subjected to root-dip inoculation with *V. dahliae*. Phenotypic observations were recorded at 15 days post-inoculation (dpi). Compared with the Mock and TRV::*00* empty vector control groups, the TRV::*GhAOS1*-silenced plants exhibited typical VW symptoms, including obvious leaf wilting, yellowing, necrosis, and even defoliation, with a significantly elevated disease index. Meanwhile, fungal biomass quantification revealed that the amount of *V. dahliae* in silenced plants was significantly higher than that in control plants (Figure 2A,B). These results demonstrate that silencing *GhAOS1* significantly compromises cotton resistance to *V. dahliae*, confirming that *GhAOS1* positively regulates cotton defense against VW.

To further validate the function of *GhAOS1*, a transgenic *Arabidopsis* line overexpressing *GhAOS1* was generated. Based on positive DNA identification in the T_1_ generation and high expression levels of the transgene detected by RT-qPCR in the T_2_ generation, three homozygous T_3_ transgenic lines were selected (OE-*GhAOS1*-2/3/6) for phenotypic analysis (Figure S2A,B). At 14 dpi, OE-*GhAOS1*-2/3/6 plants exhibited significantly enhanced resistance to *V. dahliae* compared to the WT controls (Figure 2D). Consistent with this observation, the DIs of the overexpression lines were significantly lower than those of WT (Figure 2E), accompanied by a marked reduction in fungal biomass in the roots (Figure 2F). Collectively, these results demonstrate that heterologous expression of *GhAOS1* confers enhanced resistance to *V. dahliae* in *Arabidopsis*.

### 2.3. Silencing of GhAOS1 in Cotton Reduces the JA Content in Cotton

**Figure 3:**
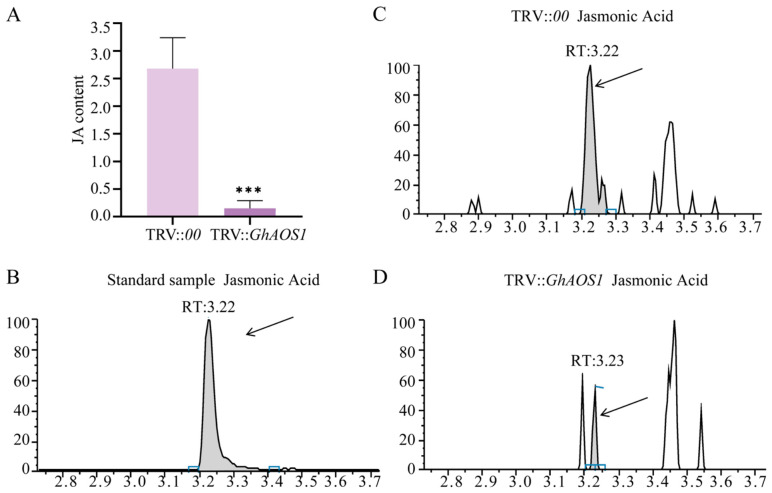
JA content in TRV::*00* and TRV::*GhAOS1* cotton plants. (**A**) JA content in TRV::*00* and TRV::*GhAOS1* plants. (**B**) Chromatographic diagram of JA standard. (**C**) Liquid chromatography detection map of JA in TRV::*00* plants. (**D**) Liquid chromatography detection map of JA in TRV::*GhAOS1* plants. Arrows indicate the peak of JA at the RT of 3.22 min. Data were expressed as mean ± SD, *n* = 3 (*** *p* < 0.001, Student‘s *t*-test).

Endogenous JA levels in cotton plants from the TRV::*00* control group and TRV::*GhAOS1*-silenced group were determined using ultra-high performance liquid chromatography coupled with high-resolution Orbitrap mass spectrometry (UHPLC-Q Exactive; Thermo Fisher Scientific, USA). Chromatographic analysis revealed that the retention time (RT) of the JA standard was 3.22 min, with the JA peak in TRV::*00* control leaves eluting at the identical retention time (RT = 3.22 min) (Figure 3B,C). In contrast, the JA signal peak in the TRV::*GhAOS1*-silenced group was markedly decreased (RT = 3.23 min) (Figure 3D). Quantitative results further demonstrated that the JA content in the TRV::*00* control group was 2.69 ng/g, whereas the TRV::*GhAOS1*-silenced group exhibited a JA content of merely 0.15 ng/g, representing a significant decrease compared with the control (Figure 3A). These findings confirm that *GhAOS1* silencing substantially suppresses JA biosynthesis in cotton, indicating that *GhAOS1* participates in the regulation of cotton resistance against *V. dahliae* through positive modulation of JA biosynthesis.

### 2.4. GhAOS1-Enabled Resistance to V. dahliae Involves JA Metabolism

**Figure 4:**
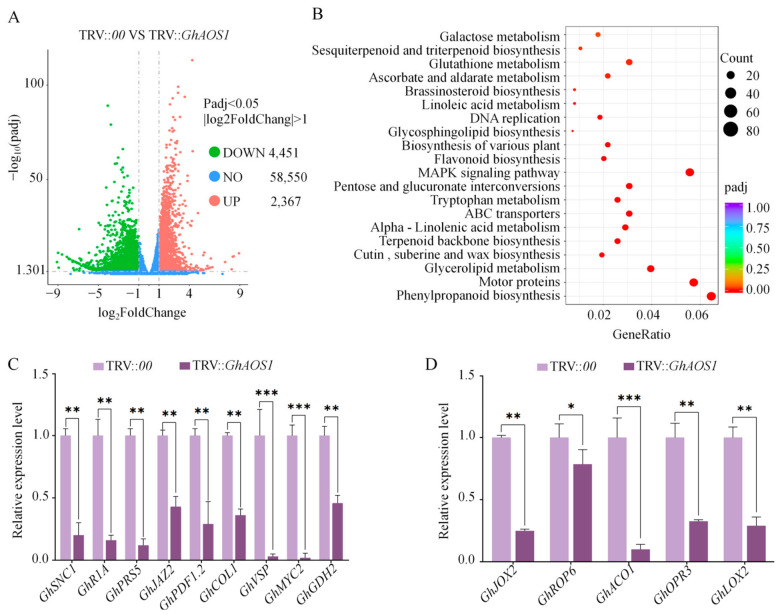
Transcriptome analysis of *GhAOS1*-silenced (VIGS_*GhAOS1*). (**A**) Volcano plot of the 6818 DEGs between TRV::*GhAOS1* and TRV::*00*. (**B**) Kyoto Encyclopedia of KEGG pathway enrichment analysis of the DEGs between TRV::*GhAOS1* and TRV::*00*. (**C**) The expression of plant immune defense-related genes. (**D**) The expression of JA biosynthesis and metabolism-related genes. Data were expressed as mean ± SD, *n* = 3 (*, *p* < 0.05, **, *p* < 0.01, ***, *p* < 0.001, Student’s *t*-test).

To decipher transcriptomic alterations following *GhAOS1* silencing in cotton, RNA sequencing was performed on *V. dahliae*-induced plants from the TRV::*00* control and TRV::*GhAOS1*-silenced groups. Volcano plot analysis identified a total of 6818 DEGs, comprising 2367 upregulated genes (red dots) and 4451 downregulated genes (green dots) (Figure 4A). KEGG enrichment analysis further revealed that these DEGs were significantly enriched in pathways closely associated with disease resistance and JA biosynthesis, including flavonoid biosynthesis, phenylpropanoid biosynthesis, α-linolenic acid metabolism, and linoleic acid metabolism, elucidating the regulatory network through which *GhAOS1* silencing modulates cotton defense responses against VW via multiple metabolic pathways (Figure 4B).

To validate the central role of *GhAOS1* in cotton disease resistance regulation, qRT-PCR was conducted on plant immunity-related genes, including *GhSNC1*, *GhR1A*, and *GhPSS5.* The results demonstrated that the expression levels of these defense-related genes were significantly downregulated in TRV::*GhAOS1*-silenced plants compared with the TRV::*00* control group, suggesting that *GhAOS1* silencing may compromise cotton defense capacity through the suppression of defense gene expression (Figure 4C).

To determine whether *GhAOS1* regulates cotton resistance to *V. dahliae* through the JA signaling pathway, expression analysis was further performed on genes involved in JA biosynthesis and metabolism, such as *GhJOX2*, *GhROP6*, *GhAOC1*, *GhOPR3*, and *GhLOX2*. The qRT-PCR results indicated that the expression levels of these key JA pathway genes, including *GhROP6* and *GhAOC1*, were significantly reduced in TRV::*GhAOS1*-silenced plants (Figure 4D). Collectively, these findings demonstrate that *GhAOS1* participates in cotton defense against *V. dahliae* through positive regulation of the JA signaling pathway and downstream defense gene expression.

### 2.5. GhWRKY70 Directly Interacts with the Promoter of GhAOS1

**Figure 5:**
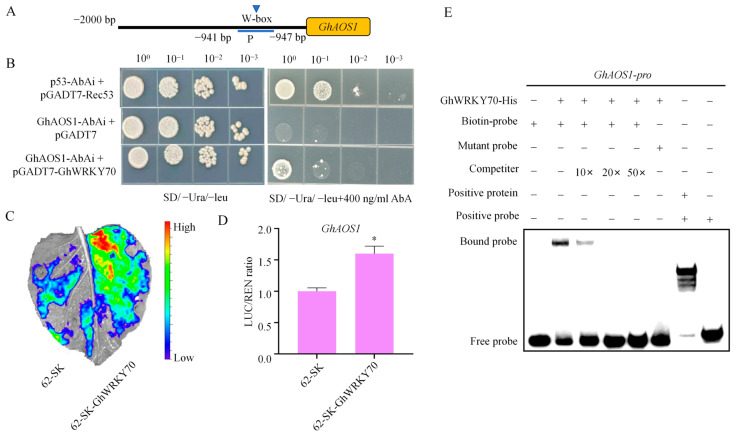
Interaction detection between GhWRKY70 and the GhAOS1 promoter. (**A**) Schematic diagram of the *GhAOS1* promoter region. The predicted W-box element located at −941 to −947 bp upstream of the transcription start site. (**B**) The yeast one-hybrid assay. (**C**) Transient expression assays in *N. benthamiana.* (**D**) Transient expression assay of promoter activity. (**E**) EMSA assay confirming the interaction between GhWRKY70 and the promoter of *GhAOS1*. “W-box” refers to the motif “TTGACC”, “W-box_Mut_” refers to the motif “AAAAA”. Data were expressed as mean ± SD, *n* = 3 (*, *p* < 0.05, Student’s *t*-test).

Based on the WRKY transcription factor binding element “W-box” predicted in the *GhAOS1* promoter sequence (Figure 5A), and the significantly downregulated gene GhWRKY70 screened from the *V. dahliae*-induced TRV::*GhAOS1* transcriptome, we hypothesized that *GhAOS1* may serve as a potential target gene regulated by GhWRKY70. A Y1H assay was performed to investigate the interaction between GhWRKY70 and the *GhAOS1* promoter. The autoactivation assay of GhAOS1-pAbAi showed that the self-activation of *GhAOS1* was suppressed in SD/-Ura medium containing 400 ng/mL AbA (Figure S3). In SD/-Ura/-Leu medium, the positive control, those cotransformed pGADT7-GhWRKY70 or pGADT7 (negative controls) with a bait fragment containing the W-box (negative control), grew normally. However, after supplementation with 400 ng/mL AbA, the negative control failed to grow, while the growth of the positive control and bait–prey transformants survived (Figure 5B). GhWRKY70 strongly activated GhAOS1 promoter activity, as evidenced by an increased LUC/REN ratio in the presence of GhWRKY70 (Figure 5C,D). The EMSA results demonstrated that the transcription factor GhWRKY70 directly binds to the W-box element in the promoter region of the *GhAOS1* gene, but not to the probes that carry the mutated W-box element (Figure 5E), indicating that GhWRKY70 may participate in the regulation of the JA biosynthesis pathway by modulating the transcription of *GhAOS1*.

### 2.6. Silencing of GhWRKY70 in Cotton Confers Sensitivity to V. dahliae via JA Metabolism

**Figure 6:**
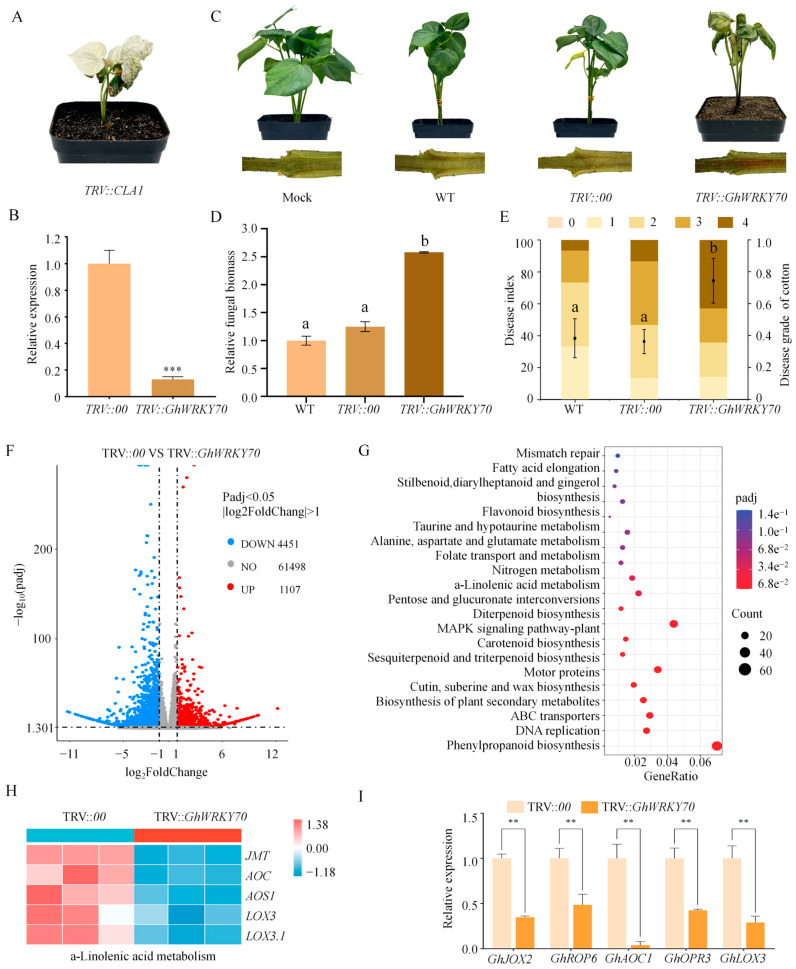
GhWRKY70 acted as a positive regulator in cotton defense against *V. dahliae.* (**A**) Photobleaching phenotype in TRV::*CLA1*-positive control plants. (**B**) Silencing efficiency of *GhWRKY70* in VIGS plant, determined by qRT-PCR. (**C**) Disease symptoms of WT, TRV::*00* (control), and *GhWRKY70*-silenced (TRV::*GhWRKY70*) plants after inoculation with *V. dahliae*. (**D**) DI of WT, TRV::*00*, and TRV::*GhWRKY70* plants at 14 dpi. Rated on a 0–4 disease severity scale. (**E**) Fungal biomass quantified by qRT-PCR in stem tissues at 14 dpi. *GhUBQ7* was used as the endogenous reference gene. Data represent mean ± SE of three biological replicates. (**F**) Volcano plot of the 5,558 DEGs between VIGS_*GhWRKY70* and WT. (**G**) Kyoto KEGG pathway enrichment analysis of the DEGs between VIGS_*GhWRKY70* and WT. (**H**) Heatmap of the expression of key genes involved in the JA signaling pathway. (**I**) The expression of JA biosynthesis and metabolism-related genes. *GhUBQ7* is the internal reference. Different letters indicate significant differences at *p* < 0.05, as determined using ANOVA. Data were expressed as mean ± SD, *n* = 3 (**, *p* < 0.01, ***, *p* < 0.001, Student’s *t*-test).

To clarify the function of *GhWRKY70* in cotton resistance to *V. dahliae*, a VIGS system was used to silence *GhWRKY70*. Successful silencing was verified by the photobleaching phenotype in TRV::*CLA1* plants (Figure 6A) and significant downregulation of *GhWRKY70* expression (Figure 6B). Compared to WT, *GhWRKY70*-silenced plants exhibited more severe disease symptoms, including wilting, yellowing, necrosis, and leaf abscission, along with enhanced vascular browning (Figure 6C). DI and fungal biomass were significantly higher in *GhWRKY70*-silenced plants than in the WT controls (Figure 6D,E). These results demonstrated that *GhWRKY70* acted as a positive regulator of cotton resistance to *V. dahliae*.

RNA-seq was conducted on TRV::*00* and TRV::*GhWRKY70* plants inoculated with *V. dahliae*. A total of 1107 upregulated and 4451 downregulated genes were identified (Figure 6F). KEGG enrichment analysis revealed that the DEGs were primarily enriched in pathways closely associated with disease resistance and JA biosynthesis, including phenylpropanoid biosynthesis, a-Linolenic acid metabolism, MAPK signaling pathway-plant, and ABC transporters (Figure 6G). Specifically, compared to TRV::*00*, a-Linolenic acid metabolism genes were significantly downregulated in TRV::*GhWRKY70* plants (Figure 6H). RT-qPCR further confirmed that the expression levels of JA signaling pathway-related genes (*GhJOX2*, *GhROP6*, *GhAOC1*, *GhOPR3*, and *GhLOX2*) were significantly lower compared to the control (TRV::*00*) (Figure 6I). Our study demonstrated that *GhWRKY70* contributes to cotton susceptibility to *V. dahliae* through the regulation of the JA signaling pathway.

## 3. Discussion

Cytochrome P450 monooxygenases constitute one of the largest and most functionally diverse enzyme superfamilies in nature [28,29]. In plants, members of this family participate in the biosynthesis of a wide range of secondary metabolites, including alkaloids, terpenoids, phenylpropanoids, and lipid-derived compounds such as JA [29,30]. These metabolites contribute to plant defense against pathogens and herbivores. Within the P450 superfamily, the PLN02648 subfamily includes AOS, which catalyzes the first irreversible and rate-limiting step in jasmonic acid biosynthesis from α-linolenic acid-derived hydroperoxides [31,32]. Chloroplasts are important sites of lipid metabolism and reactive oxygen species production and are the primary location for the formation of α-linolenic acid-derived hydroperoxy fatty acids such as 13-HPOT. Domain analysis showed that GhAOS1 contains the conserved PLN02648 (AOS) and CYP74-Ae domains, which are characteristic of AOS enzymes. Consistently, subcellular localization confirmed that GhAOS1 is localized in the chloroplast (Figure 1D), the well-established site of JA biosynthesis [33]. Homology modeling further revealed that GhAOS1 possesses a typical catalytic pocket characteristic of cytochrome P450 enzymes. Several predicted key residues (including F142, N326, V393, and C476) are likely involved in substrate recognition, stabilization of reaction intermediates, or maintenance of the catalytic microenvironment (Figure 1B). Notably, cysteine (C476) is predicted to act as the fifth ligand coordinating the heme iron, a conserved feature of many P450 enzymes that is essential for maintaining catalytic activity and proper enzymatic function [34].

Plants activate multiple defense responses upon pathogen invasion, including ROS bursts, cell wall reinforcement, accumulation of pathogenesis-related proteins, and reprogramming of hormone signaling networks [35,36,37]. Jasmonic acid and its derivatives are widely recognized as essential phytohormones for plant defense against necrotrophic and hemibiotrophic pathogens [38,39,40]. As a key rate-limiting enzyme in JA biosynthesis, AOS has been widely reported to contribute to plant disease resistance [31,41,42]. AOS function has been most extensively studied in tomato, where disruption or VIGS-mediated silencing of *SlAOS* impairs JA biosynthesis and increases susceptibility to pathogens such as *Pseudomonas syringae* and *Botrytis cinerea*, highlighting the critical role of AOS-mediated JA production in plant defense [31,43]. Similarly, this study demonstrated through tissue-specific expression analysis, cotton VIGS assays, and overexpression in transgenic *Arabidopsis* that *GhAOS1* positively regulates resistance to VW in cotton (Figure 2). This finding is consistent with our transcriptome data, in which silencing *GhAOS1* resulted in the downregulation of a large number of genes associated with the jasmonic acid (JA) pathway and disease resistance.

Further transcriptome sequencing showed that DEGs were significantly enriched in phenylpropanoid biosynthesis, flavonoid biosynthesis, and α-linolenic acid metabolism (Figure 4B). The phenylpropanoid pathway is a central hub for the production of diverse plant defense compounds, and its product lignin serves as a crucial barrier that reinforces the cell wall and prevents the physical invasion of pathogens [27,44,45]. Flavonoids possess broad-spectrum antimicrobial activities and can also function as signaling molecules regulating immune responses [46]. Meanwhile, α-linolenic acid metabolism represents the direct precursor pathway for JA biosynthesis [47]. These results directly link *GhAOS1* with several classical disease resistance metabolic pathways, suggesting that *GhAOS1* regulates JA biosynthesis and subsequently influences multiple downstream defense pathways, including phenylpropanoid metabolism, ultimately coordinating the overall disease resistance response in cotton.

In *GhAOS1*-silenced plants, the expression levels of several known disease resistance-related genes were significantly downregulated. For example, the disease resistance regulator *GhSNC1*, whose constitutive activation can confer broad-spectrum disease resistance [48]. GhGDH2 regulates cotton resistance to VW through the JA and SA signaling pathways [49]. *GhROP6* belongs to the ROP small GTPase family, and GhROP6 may be involved in the resistance of cotton to VW through JA synthesis and signaling pathway and lignin synthesis [50]. Yeast two-hybrid and BiFC assays showed that GhJAZ2 interacts with the NBS-LRR disease resistance protein GhR1A, suggesting that JA signaling may crosstalk with ETI in cotton defense responses [51]. The overexpression of GhJAZ2 in cotton impairs sensitivity to JA, decreases the expression level of JA-response genes (*GhPDF1.2* and *GhVSP*), and enhances the susceptibility to *V. dahliae* and insect herbivory [51]. In this study, these defense-related proteins were significantly downregulated in the *GhAOS1*-silenced plants (Figure 4C). Meanwhile, the expression levels of several key genes in the JA signaling pathway were also markedly reduced (Figure 4D). For example, GhAOC1 (Allene Oxide Cyclase 1), which acts immediately downstream of AOS and catalyzes the formation of the key JA biosynthetic intermediate 12-oxo-phytodienoic acid (OPDA), was significantly downregulated. GhOPR3 is responsible for reducing OPDA to the direct precursor of JA [52]. GhLOX2, a 13-lipoxygenase, participates in the initial step of JA biosynthesis by catalyzing the conversion of linolenic acid to 13-hydroperoxylinolenic acid, thereby positively regulating cotton tolerance to VW through the JA-mediated pathway [53]. More importantly, the JA content in the silenced plants decreased by approximately 94% compared with the control plants, dropping from 2.69 ng/g to 0.15 ng/g (Figure 3A–D). These results further demonstrate that *GhAOS1* positively regulates cotton resistance to VW. The loss of *GhAOS1* markedly weakened the activation of these key defense regulatory factors mediated by JA signaling. By maintaining JA metabolic homeostasis and activating downstream defense-related gene expression, *GhAOS1* ultimately enhances the ability of cotton plants to resist *V. dahliae* infection.

Transcriptome analysis of *GhAOS1*-silenced plants revealed that *GhWRKY70* was significantly downregulated. The WRKY transcription factor family is one of the largest transcriptional regulatory families in plants and plays a central role in responses to both biotic and abiotic stress [54]. Members of this family regulate downstream gene expression by specifically recognizing W-box elements ((T)(T)TGAC(C/T)) in the promoter regions of target genes, thereby participating in plant defense responses against a wide range of pathogens, including fungi, bacteria, oomycetes, and viruses [55]. Within the plant immune regulatory network, WRKY transcription factors interact with MAPK signaling cascades, plant hormone signaling pathways, and other transcription factors, forming complex regulatory networks that precisely control plant defense responses [56]. The disease resistance function of WRKY70 has been extensively studied in other plant species, showing both conserved and diversified roles across different plant–pathogen systems. For example, overexpression of AtWRKY70 activates salicylic acid (SA)-responsive genes and enhances resistance to the hemibiotrophic pathogen *Pseudomonas syringae*, while simultaneously increasing susceptibility to the necrotrophic pathogen *Alternaria brassicicola* [57]. In addition, AtWRKY70 cooperates with its homolog WRKY54 to negatively regulate SA biosynthesis; the wrky54/wrky70 double mutant exhibits elevated SA levels, increased hydrogen peroxide accumulation, and enhanced cell wall defenses, thereby improving resistance to necrotrophic pathogens such as *Pectobacterium carotovorum* and *Botrytis cinerea* [58]. Based on the predicted WRKY transcription factor binding elements in the *GhAOS1* promoter, together with the known regulatory roles of WRKY70 in plant disease resistance and JA signaling pathways, our results are consistent with the finding that silencing of GhWRKY70 in cotton resulted in decreased expression of key genes involved in JA biosynthesis (Figure 6H,I). Furthermore, Y1H, LUC, and EMSA confirmed that GhWRKY70 functions as a transcriptional activator that directly binds to and activates the *GhAOS1* promoter (Figure 5B–E). Moreover, VIGS of GhWRKY70 demonstrated that silencing this gene significantly reduced cotton resistance to *V. dahliae*, confirming that GhWRKY70 acts as a positive regulator in the cotton defense response against VW (Figure 6A–E). This finding not only refines the transcriptional regulatory network underlying cotton resistance to VW but also provides direct evidence for the role of WRKY transcription factors in JA-mediated plant immunity, offering an important theoretical foundation for future molecular breeding strategies aimed at improving cotton resistance to VW.

## 4. Materials and Methods

### 4.1. Plant Materials

*Gossypium hirsutum* cv. Coker 312, *Arabidopsis thaliana* (ecotype Columbia-0), and *Nicotiana benthamiana* were obtained from the National Key Laboratory of Agricultural Microbiology, Biotechnology Research Institute, Chinese Academy of Agricultural Sciences, Beijing, China. Plants were maintained in a greenhouse under a 16 h light/8 h dark cycle at 25 °C and irrigated weekly with Hoagland’s nutrient solution.

### 4.2. Bioinformatics Analysis

Protein sequences highly homologous to GhAOS1 from multiple species, including *Gossypium hirsutum*, *Gossypium barbadense*, *Theobroma cacao*, *Populus trichocarpa*, *Vitis vinifera*, *Solanum tuberosum*, *Solanum lycopersicum*, *Arabidopsis thaliana*, *Glycine max*, *Cajanus cajan*, and *Oryza sativa* were retrieved from the NCBI database (https://www.ncbi.nlm.nih.gov/). Multiple sequence alignment and phylogenetic tree construction were performed in MEGA11 using the ClustalW algorithm and the neighbor-joining method, respectively. The three-dimensional structure of GhAOS1 was predicted using SWISS-MODEL (https://swissmodel.expasy.org/), while homology modeling and active-site prediction were conducted using the PlayMolecule online server (https://playmolecule.ai/, accessed on 1 May 2026).

### 4.3. RT-qPCR Analysis

The highly virulent defoliating *V. dahliae* strain V991 was cultured on potato dextrose agar (PDA) at 25 °C for 7–10 days, and the colonies were then transferred to liquid complete medium (CM) at 25 °C with shaking at 200 rpm for 2–3 days for inoculum preparation [59,60]. When cotton seedlings reached the three-true-leaf stage, their roots were immersed in V991 conidial suspension for 0, 0.5, 1.0, 2.0, 4.0, 8.0, and 12.0 h. Subsequently, RNA was extracted from root, stem, and leaf tissues and reverse-transcribed into cDNA. The cotton housekeeping gene *GhUBQ7* (LOC107925174) served as the internal reference gene. qRT-PCR amplification was performed using the primer pairs GhUBQ7-RT-F/R and GhAOS1-qRT-F/R (Table S1), with three technical replicates for each sample. After the reaction, the expression level of the target gene was calculated using the 2^−ΔΔCt^ method [61] based on the Ct values of both the target and reference genes.

### 4.4. Subcellular Localization

The *GhAOS1* full-length ORF was amplified and cloned into the *Bam*H I and *Eco*R I sites of the pYBA1132-eGFP expression vector. The recombinant plasmids were transformed into *A. tumefaciens* strain GV3101 (pSoup-19) and transiently expressed in tobacco plants. Tobacco leaf abaxial epidermal cells were co-infiltrated with pYBA1132-GhAOS1 and the empty vector control pYBA1132, respectively. After 48 h post-inoculation (hpi), GFP fluorescence was observed using an LSM980 confocal laser scanning microscope (Zeiss, Oberkochen, Germany). GFP was excited at 488 nm and detected at 500–530 nm; chlorophyll autofluorescence was excited at 561 nm and detected at 650–750 nm. Chloroplast localization was determined by evaluating the co-localization of GFP signals with chlorophyll autofluorescence.

### 4.5. Cotton VIGS

The specific regions of *GhAOS1* and *GhWRKY70* amplified from the cDNA of cv. Coker 312 were cloned into the pTRV2 vector using *Eco*R I and *Bam*H I restriction sites. The primer sequences were listed in Table S1. The chloroplastos alterados 1 (*CLA1*) gene was used as a marker to monitor silencing reliability. Cotton VIGS was performed according to a previously described procedure [62]. Two weeks after injection, RNA was extracted from newly grown leaves to detect the expression of target genes. More than 30 seedlings were used for each analysis. Plants that were successfully silenced were selected for the subsequent *V. dahliae* infection assay and other assays. All VIGS assays were performed three times independently.

### 4.6. Generation of Transgenic Arabidopsis

The *GhAOS1* full-length ORF was amplified and ligated into the *Bam*H I and *Sal* I sites of pCAMBIA2300-GFP to obtain the pCAMBIA2300-GhAOS1 overexpression vector. The plasmid was introduced into *Agrobacterium tumefaciens* strain GV3101, and transgenic *Arabidopsis* plants were subsequently generated using the floral dip method [63]. Transgenic positive plants were preliminarily identified by PCR and RT-qPCR, with *AtActin2* as the internal reference gene.

### 4.7. Plant Inoculation and Disease Assay

To study the performance of silent/transgenic materials in response to *V. dahliae*, three-leaf stage cotton seedlings and four-week-old *Arabidopsis* plants were inoculated with *V. dahliae* strain V991. The inoculated silent plants and transgenic plants were grown in a greenhouse at the appropriate temperatures depending on the plant type (25 °C for cotton and 20 °C for *Arabidopsis*). The plant disease indices and disease ratios were calculated as previously described [64,65].

Fungal biomass was quantified by extracting genomic DNA from inoculated *Arabidopsis* and cotton plants. The ITS1 and ITS2 regions of the *V. dahliae* ribosomal RNA gene (accession Z29511) were amplified using specific primers Vd-ITS-F/R, with *AtUBQ10* serving as the internal reference gene (Table S1).

### 4.8. Transcriptome Sequencing

The plants of TRV::*GhAOS1* and TRV::*00* plants were collected 2 weeks after VIGS injection. Total RNA was extracted using the RNAprep Pure Plant Plus Kit (TIAGEN, Beijing, China) for RNA sequencing. Each treatment included three independent samples. The quality of the extracted RNA was assessed using an Agilent 2100 Bioanalyzer (Agilent, Santa Clara, CA, USA). mRNA was enriched with oligo(dT) magnetic beads, fragmented, and used for library construction with the NEBNext Ultra RNA Library Prep Kit for Illumina. Libraries were sequenced on the Illumina NovaSeq 6000 platform (Illumina, San Diego, CA, USA). Raw reads were filtered with Fastp v0.19.7 (Haplox, Shenzhen, China) to remove the reads that contained adapters, poly-N sequences, and low-quality reads (defined as those with more than 30% of bases having a quality score below 30). The resulting clean reads were mapped to the cotton reference genome on CottonGen (https://www.cottongen.org/species/Gossypium_hirsutum/nbi-AD1_genome_v1.1, accessed on 25 May 2024). Differentially expressed genes (DEGs) were identified using DESeq with thresholds of |log2 (FoldChange)| ≥ 1 and padj ≤ 0.05, and gene expression levels were normalized as FPKM values. Gene Ontology (GO) and the Kyoto Encyclopedia of Genes and Genomes (KEGG) were used for functional annotation and enrichment analysis.

### 4.9. Y1H Assay

The full-length coding sequence of GhWRKY70 was amplified and cloned into the pGADT7 vector via *Eco*RI and *Bam*HI sites, generating the recombinant plasmid pGADT7-GhWRKY70. Meanwhile, the promoter region of the *GhAOS1* gene was amplified from cotton genomic DNA and inserted into the pAbAi bait vector after double digestion with *Sal*I and *Hin*dIII, constructing the GhAOS1Pro-pAbAi bait plasmid. The GhAOS1Pro-pAbAi bait plasmid was transformed into Y1HGold yeast competent cells using the LiAc/PEG-mediated method and plated on SD/-Ura medium, followed by incubation at 28 °C for 3 d. The transformants were screened using Insert Check Mix1 (Takara, Dalian, China) to identify positive clones. The positive transformants were then inoculated onto SD/-Ura selective plates containing different concentrations of aureobasidin A (AbA) for gradient concentration screening.

The empty AD vector and the pGADT7-GhWRKY70 recombinant plasmid were separately transformed into Y1HGold competent cells containing the GhAOS1Pro-pAbAi bait plasmid, and the transformants were plated on SD/-Leu/-Ura medium supplemented with different concentrations of AbA. Simultaneously, p53-AbAi + pGADT7-Rec53 was set as the positive control and plated on SD/-Ura/-Leu/400 ng/mL AbA medium. All plates were incubated at 28 °C for 3 d.

### 4.10. LUC Reporter System

The *GhAOS1* promoter fragment was cloned into the pGreenII 0800-LUC reporter vector via *Kpn*I and *Eco*RI double digestion, generating the ProGhAOS1-LUC recombinant vector. The coding sequence of *GhWRKY70* was amplified and inserted into the pGreenII62-SK effector vector following *Hin*d III and *Bam*H I double digestion, yielding the 62-SK-GhWRKY70 recombinant vector. Both recombinant plasmids were individually transformed into *A. tumefaciens* strain GV3101. Positive clones were verified by bacterial PCR, and the bacterial suspensions were adjusted to OD_600_ = 1.0. The effector and reporter vector suspensions were mixed at a 1:1 volume ratio and infiltrated into the abaxial epidermis of tobacco leaves. Six independent leaves were injected for each treatment, with 62-SK + ProGhAOS1 serving as the control group and 62-SK-GhWRKY70 + ProGhAOS as the experimental group. Firefly luciferase (LUC) fluorescent signals were visualized using a plant in vivo imaging system. We used a Dual-Luciferase kit (Promega, Madison, WI, USA) for transient expression analysis to detect reporter activity. The Renilla (REN) luciferase gene was used as an internal control. The ratio of LUC/REN was calculated as an indicator of the final transcriptional activity.

### 4.11. EMSA

EMSA was performed using the LightShift Chemiluminescent EMSA Kit (Thermo Fisher Scientific, Waltham, MA, USA). The recombinant GhWRKY70-His protein was incubated with biotin-labeled probes containing the W-box (TTGACC/T) element in a binding buffer for 20 min at room temperature. For the competition assay, a 10-fold or 20-fold molar excess of unlabeled probe was added to the reaction mixture before incubation. The DNA–protein complexes were separated on a 6% native polyacrylamide gel in 0.5×TBE buffer at 100 V. The gel was transferred to a positively charged nylon membrane and cross-linked under UV light. Biotin-labeled DNA was detected using a chemiluminescent nucleic acid detection module according to the manufacturer’s instructions.

### 4.12. JA Content Determination

Endogenous JA levels were quantified in TRV::*GhAOS1*-silenced cotton plants and TRV::*00* control plants using ultra-high performance liquid chromatography coupled with high-resolution mass spectrometry (UHPLC-Q Exactive, Thermo Fisher Scientific, Waltham, MA, USA). Fresh leaf tissues (0.5 g) were ground into fine powder in liquid nitrogen and transferred to a 2 mL centrifuge tube. Pre-cooled 50% acetonitrile aqueous solution (1 mL) was added to the tube, and the mixture was vortexed for 30 s and incubated at 4 °C for 2 h for extraction. The mixture was centrifuged at 12,000 rpm for 10 min at 4 °C, and the supernatant was collected; the extraction step was repeated once, and the two supernatants were combined. The combined supernatant was concentrated to near dryness under a stream of nitrogen, reconstituted with 0.2 mL of methanol, and filtered through a 0.22 μm organic filter membrane into a sample vial with an insert for instrumental analysis. Chromatographic separation was performed on a Waters HSS T3 column (50 mm × 2.1 mm, 1.8 μm) using a Thermo Q Exactive high-resolution mass spectrometer (Thermo Fisher Scientific, Waltham, MA, USA), with samples maintained at 4 °C in the autosampler. JA was quantified by external standard calibration using Trace Finder software 5.1 (Thermo Fisher Scientific, Waltham, MA, USA). The JA content (ng/g) was calculated as: (C × V)/M × 1000, where C is the sample concentration (ng/mL), V is the extraction volume (mL), and M is the sample weight (mg).

### 4.13. Statistical Analysis

All data were presented as the mean ± standard deviation (SD). Statistical analyses were conducted using GraphPad Prism 8.0 (Dotmatics, San Diego, CA, USA). Statistical significance was determined using Student’s *t*-test or ANOVA, followed by Tukey’s or Dunnett’s multiple comparison test.

## 5. Conclusions

This study demonstrates that *GhAOS1* positively regulates cotton resistance to *V. dahliae* by modulating jasmonic acid biosynthesis. GhAOS1 is induced by pathogen infection and localized in chloroplasts. Silencing *GhAOS1* significantly reduces endogenous JA levels and suppresses defense-related gene expression, leading to increased susceptibility to VW. Mechanistically, the transcription factor GhWRKY70 directly activates *GhAOS1* transcription by binding to the W-box element in its promoter. Silencing GhWRKY70 similarly weakens JA signaling and cotton resistance to *V. dahliae*. Together, these results uncover a GhWRKY70-*GhAOS1* regulatory module that coordinates JA-mediated defense responses in cotton.

## Data Availability

All data are included in the manuscript and the Supporting Information. The RNA-seq datasets generated in this study have been deposited in the NCBI (https://www.ncbi.nlm.nih.gov/) database under accession number PRJNA1443982.

## Supplementary Materials

The following supporting information can be downloaded at: https://www.mdpi.com/article/10.3390/ijms27114713/s1.

## Abbreviations

The following abbreviations are used in this manuscript:

DEGs	Differentially expressed genes
Y1H	Yeast one-hybrid
LUC	Dual-luciferase
EMSA	Electrophoretic mobility shift assays
VIGS	Virus-induced gene silencing
RT-qPCR	Real-time quantitative PCR
