# Supplementary material for: The GhWRKY70-GhAOS1 Axis Integrates Jasmonate Pathway Signaling to Regulate Cotton Immunity Against Verticillium dahliae
Source: Int J Mol Sci. 2026 May 23;27(11):4713. doi: 10.3390/ijms27114713 (PMC13256778; doi:10.3390/ijms27114713)
Supplement: Supplementary file 1 [file ijms-27-04713-s001.zip › ijms-4306865-supplementary.pdf]

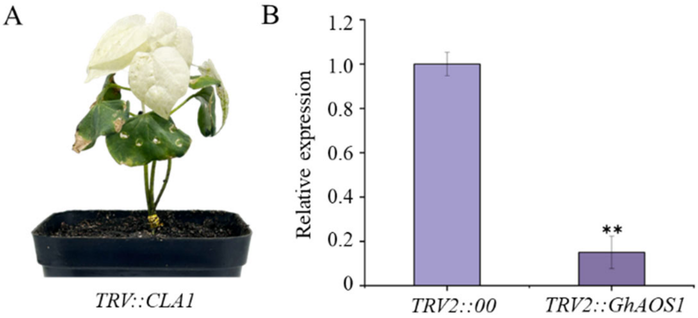

Figure S1. Detection of *GhAOS1* silencing efficiency. (A) The positive control of VIGS in which the cotton *CLA1* gene was silenced to produce the albino phenotype. (B) *GhAOS1* expression in TRV::*GhAOS1* and control plants, *GhUBQ7* was used as the internal reference gene. Relative expression levels were calculated using the $2^{-\Delta\Delta Ct}$ method. Asterisks indicate significant differences, as determined using Student's *t*-test; **, $P<0.01$.

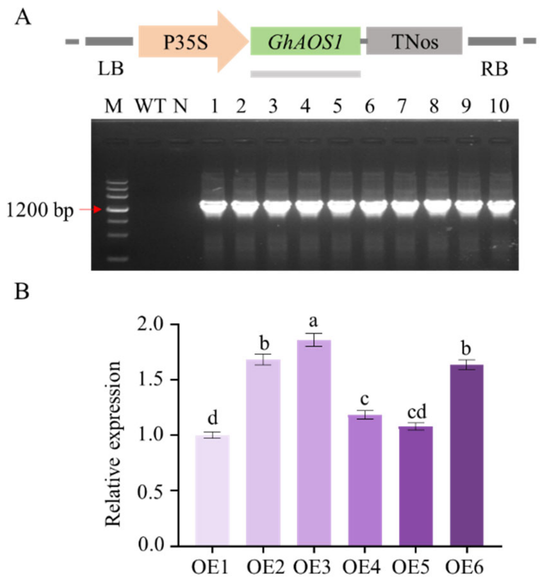

Figure S2. Analysis of transgenic *GhAOS1 Arabidopsis* lines. (A) Schematic diagram of the construction of the *GhAOS1* transgenic *Arabidopsis* vector and PCR identification of *GhAOS1* positive plants in *Arabidopsis*. (B) RT-qPCR analysis of *GhAOS1*-positive *Arabidopsis* plants. Relative expression levels were calculated using the $2^{-\Delta\Delta Ct}$ method. Different letters indicate significant differences at $P<0.05$.

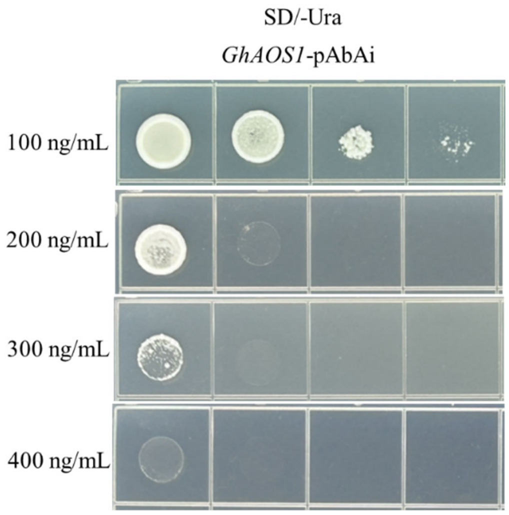

Figure S3. Autoactivation assay of *GhAOS1*-pAbAi.

Table S1 Primer list

Primer	Primer sequence (5’-3’)
QGhUBQ7-F	GCAAGTGTTGGGTTCAAAGCTGGTG
QGhUBQ7-R	CCAGGTTGAGGAGTTACTCGGAATGCTG
QVd-ITS-F	GTGAGTAAGGTTACCGAATTC
QVd-ITS-R	CGTGAGCTCGGTACCGGATCC
QGhAOS1-F	TCTACAACCAAGGCCGAGAC
QGhAOS1-R	TGGTCCGAAACACTGTCGAG
GhAOS1-1132-F	cgctctagaactagtggatccATGGCATCTTCTTCTTTACCTTTTACC
GhAOS1-1132-R	gataagcttgatatcgaattcAAAACTAGCTCTCTTCAAGGATGTGA
GhAOS1-vigs-F	tctgtgagtaaggttaccgaattcAATGAGTTATTCGAAACGTTAGAGA
GhAOS1-vigs-R	acgcgtgagctcggtaccggatccGCTTCGTAGACGACCGATTTCAT
QGhPRS5-F	GCCGTGATTCATACAGTTATCCTCA
QGhPRS5-R	TTGGCTCTTACTTCCGACCATCT
QGhJAZ2-F	CCACATCAATGCCCCCTTTACTTCT
QGhJAZ2-R	TCTTGAGTTACCGTGGTGCTGAAAT
QPDF1.2-F	GCGGATGGTGATAAGATATGCGAG
QPDF1.2-R	CCATTAGGAAAACCCTCTGTCTTGC
QGhCOI1-F	CTGCGTGATGCCGTACATCG
QGhCOI1-R	CCCACGGTGTAACGTACCCA
QGhVSP-F	TTGTTCGATGAGTGGGTAATGG
QGhVSP-R	CCTGAATACTTTGATGGTTCCTTG
QGhMYC2-F	GCTCCGCCACTACCGTGCTC
QGhMYC2-R	CTCGAAGCACTTTTTTACGGTGTTC
QGhGDH2-F	GGTCTTGGGCATCAAGGCTCAT
QGhGDH2-R	GGCACACGGGATGAGAACATCA
QGhJOX2-F	TCACTGCCCTCATCTTGCAG
QGhJOX2-R	AGACATGGCCTTGAGAAGCC
QGhROP6-F	GCTCATCTCCTACACCAGCAATAC
QGhROP6-R	CAGCAGTATCCCACAATCCAAG

QGhAOC1-F	CAACCCCTTCACTACCACTGCC
QGhAOC1-R	AGGGCTGCTTCTGTCTCTCTCG
QGhOPR3-1-F	ATGCTGTTCATGCCAAAGGAGG
QGhOPR3-1-R	TTTCTGATGTTTCCAGGGGTCG
QGhLOX2-F	GAAAGTTAGACAGCTTCCAGGGAC
QGhLOX2-R	AAGCCTCCAAAGCCAGACACCT
GhAOS1-2300-F	ggacagggtacccggggatccATGGCATCTTCTTCTTTACCTTTTACC
GhAOS1-2300-R	caccatggtactagtgtcgacAAAACTAGCTCTCTTCAAGGATGTGA
GhAOS1-AbAi-F	cttgaattcgagctcggtaccCCCAAAAAAATAGGTCACG
GhAOS1-AbAi-R	agcacatgcctcgaggtcgacGGTTGGAATTGCTTGAGG
GhWRKY70-AD-F	gccatggaggccagtgaattcTTTCCTTTTCTCTTCCATGAATTTG
GhWRKY70-AD-R	cagctcgagctcgatggatccGGAGAAAGAGCTGTATGCA
GhAOS1-0800-F	gtcgacggtatcgataagcttcattttgtaccattttcctggtc
GhAOS1-0800-R	cgctctagaactagtggatcccttctttatggatgaatcaacacaa
WRKY70-62-FP	tcccccgggctgcaggaattcatgtcttggaatacaaaaaaagcaa
WRKY70-62-RP	tcagcgtaccgaattggtacctcaaaactgaagcaaatcatcaag
QGhWRKY70-F	TGTCCCTCTTCTCTTTTCACCT
QGhWRKY70-R	CAAGACATGTTTGCTTGGGGG
GhAOS1-Biotin probe	attaagagttagggcggtcaaactgtgaaactctt
GhAOS1-mut- Biotin probe	attaagagttagggAAAAAaaactgtgaaactctt
